# Comparison of autism spectrum disorder subtypes based on functional and structural factors

**DOI:** 10.3389/fnins.2024.1440222

**Published:** 2024-10-04

**Authors:** Shan Wang, Zhe Sun, Laura Alejandra Martinez-Tejada, Natsue Yoshimura

**Affiliations:** ^1^Department of Information and Communications Engineering, School of Engineering, Tokyo Institute of Technology, Yokohama, Japan; ^2^Graduate School of Medicine, Juntendo University, Tokyo, Japan; ^3^Faculty of Health Data Science, Juntendo University, Tokyo, Japan; ^4^Department of Computer Science, School of Computing, Tokyo Institute of Technology, Yokohama, Japan

**Keywords:** ASD subtype, Asperger’s, autism, brain pattern, fMRI, PDD-NOS, tensor decomposition

## Abstract

Autism spectrum disorder (ASD) is a series of neurodevelopmental disorders that may affect a patient’s social, behavioral, and communication abilities. As a typical mental illness, ASD is not a single disorder. ASD is often divided into subtypes, such as autism, Asperger’s, and pervasive developmental disorder-not otherwise specified (PDD-NOS). Studying the differences among brain networks of the subtypes has great significance for the diagnosis and treatment of ASD. To date, many studies have analyzed the brain activity of ASD as a single mental disorder, whereas few have focused on its subtypes. To address this problem, we explored whether indices derived from functional and structural magnetic resonance imaging (MRI) data exhibited significant dissimilarities between subtypes. Utilizing a brain pattern feature extraction method from fMRI based on tensor decomposition, amplitude of low-frequency fluctuation and its fractional values of fMRI, and gray matter volume derived from MRI, impairments of function in the subcortical network and default mode network of autism were found to lead to major differences from the other two subtypes. Our results provide a systematic comparison of the three common ASD subtypes, which may provide evidence for the discrimination between ASD subtypes.

## 1 Introduction

Autism spectrum disorder (ASD) is a series of complex neurodevelopmental disorders that may affect a patient’s social, behavioral, and communication abilities (Molnar-Szakacs et al., 2021). According to the Global Burden of Disease Study in 2019, there are approximately 28 million ASD patients worldwide (Vos et al., 2020). ASD is more common in children; however, few reports of cases in adults are found due to the lack of effective intervention or unfortunate experience in adulthood. The traditional diagnosis of ASD is mainly based on the observations of doctors and various rating scales. According to the Diagnostic and Statistical Manual of Mental Disorders, Fourth Edition (DSM-IV) (American Psychological Association, 1994), ASD can be divided into multiple subtypes, including autism, Asperger’s, pervasive developmental disorder not otherwise specified (PDD-NOS), Rett’s disorder, and childhood disintegrative disorder. However, in the Diagnostic and Statistical Manual of Mental Disorders, Fifth Edition (DSM-5) (American Psychiatric Association, 2013), Rett’s disorder and childhood disintegrative disorder are no longer considered subtypes of ASD (Oberman and Kaufmann, 2020). Regardless of the rating scale used, it is widely recognized that ASD has multiple subtypes, and disorders including autism, Asperger’s, and PDD-NOS are the basic subtypes of ASD (Brown et al., 2010; Tamura et al., 2010; Sharma et al., 2018). The basis of traditional diagnosis is to compare the behavior of a person with the typical performance described in the checklist. In other words, the experience of the doctor, rather than the biological signals of the patient, plays an important role in the diagnosis. Therefore, traditional diagnoses based on rating scales are likely to be affected by various subjective factors. This subjectivity may lead to misdiagnosis, considering the heterogeneity among patients and differences between subtypes.

With the development of medical technology, many non-invasive acquisition methods have emerged to obtain signals from the human brain, including electroencephalography (EEG), magnetic resonance imaging (MRI), and functional MRI (fMRI). These non-invasive acquisition methods provide new ideas for the study of ASD (Tuchman and Rapin, 2002; Wang et al., 2013; Pierce et al., 2021). In recent years, many ASD studies have been conducted based on fMRI, due to its capabilities of providing a type of four-dimensional data that contains spatial and temporal information of the whole brain. Some studies have attempted to illustrate the impairment in brain function of ASD patients through fMRI analysis. Haghighat et al. found differences in between-connectivity among default mode network, salience-executive network, and fronto-parietal network between ASD and healthy children (Haghighat et al., 2021). Maximo et al. observed reduced brain entropy in the prefrontal areas of children with autism (Maximo et al., 2021). Another part of the studies tried to apply the deep learning method to fMRI data and classify ASD patients and healthy controls. A long short-term memory model was proposed in 2019 with data from four sites, achieving an average accuracy of 74.8% (Dvornek et al., 2019). Soon after, a 3D convolutional neural network (CNN) was proposed in 2020 and achieved an accuracy of 66.0% with data from 2085 subjects (Thomas et al., 2020). Some new deep learning methods including graph convolutional network have also appeared in the ASD studies (Parisot et al., 2018). In addition to the functional characteristics, there have also been studies focusing on the differences in brain structure between patients with ASD and healthy controls. Yaxu et al. observed atypical development in gray matter volume (GMV) and gray matter density (Yaxu et al., 2020). Watanabe and Rees identified age-associated atypical increases in relative GMVs of the regions of auditory and visual networks and an age-related aberrant decrease in the relative GMV of the fronto-parietal network in children with ASD (Watanabe and Rees, 2016). In addition, GMV is an important feature of ASD classification (Arya et al., 2020).

Although many studies have focused on the analysis or classification of ASD, few have focused on its subtypes. Previous studies have tended to focus on the difference between single subtype and typically developing controls or other mental disorders, such as attention deficit hyperactivity disorder and schizophrenia (Chen et al., 2017; Huang et al., 2020; Shi et al., 2020). In this study, we focused on the functional and structural differences between the three common ASD subtypes: autism, Asperger’s, and PDD-NOS. For fMRI data, we applied a tensor decomposition method to capture the different brain communities in the ASD subtypes. In recent years, tensor decomposition has been used to extract the features of brain activity from fMRI data (Aggarwal and Gupta, 2019; Li et al., 2020). Resting-state fMRI dataset is a high dimension data which is a combination of brain regions, time and patients. Tensor decomposition is a good tool to extract a compressed feature set or to alleviate the joint effect of factors to analyze a certain dimension. As additional functional features, amplitude of low-frequency fluctuation (ALFF) (Wang Z. et al., 2019; Arya et al., 2020) and fractional ALFF (fALFF) (Zou et al., 2008; Itahashi et al., 2015) of the subtypes, were extracted from fMRI data, and as a representation of structural features, the gray matter volume (GMV) (Zhao et al., 2022) was extracted to evaluate the variation of the brain structures among the subtypes. Based on the three functional and one structural brain features, we aimed to provide a systematic understanding of the heterogeneity among the three subtypes, which may provide a new idea for the discrimination of ASD subtypes.

## 2 Materials and methods

**Figure 1:**
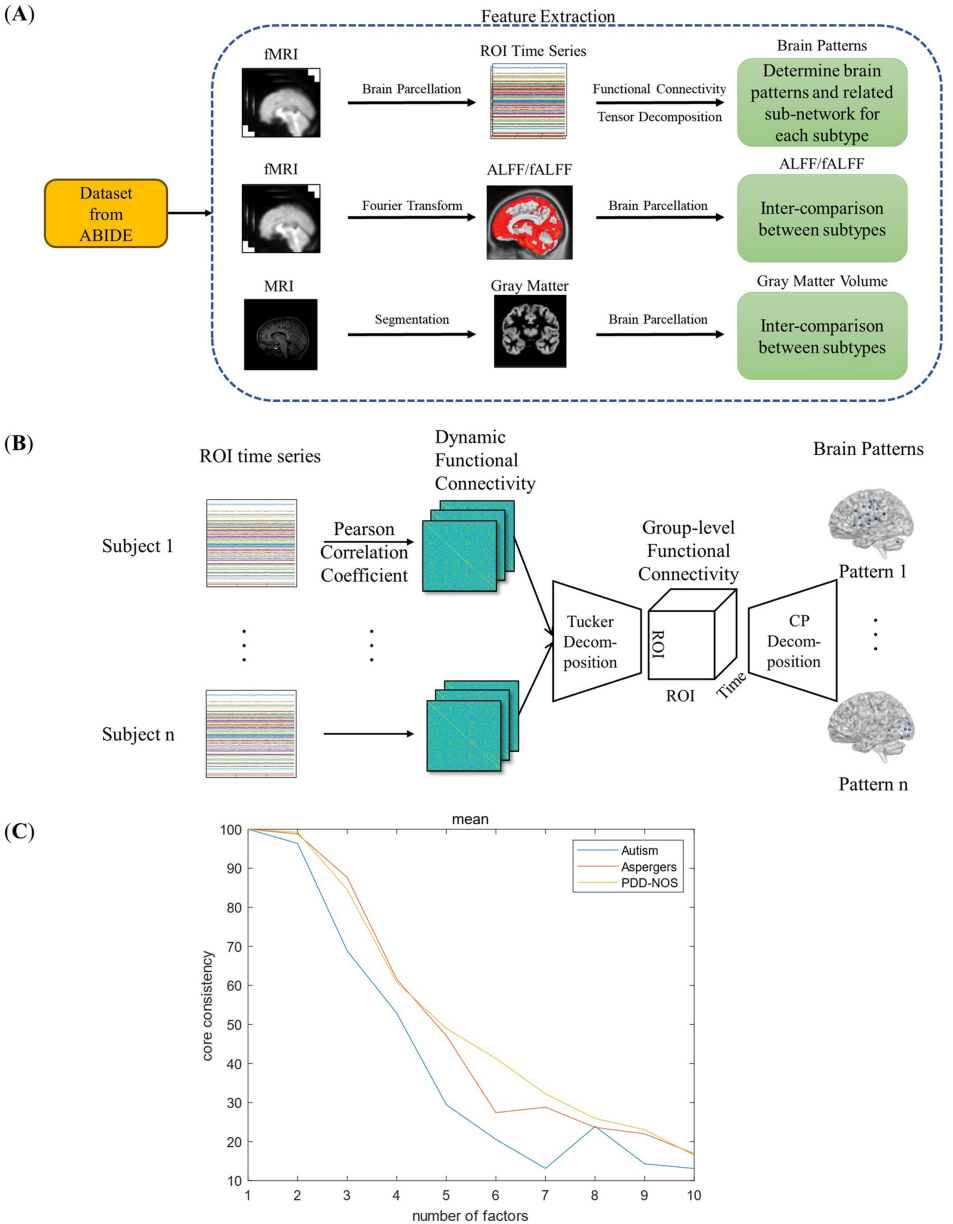
Framework of this study: **(A)** feature extraction process. **(B)** Framework of the FC-based brain pattern feature extraction. **(C)** Detection of the number of the brain patterns.

In this study, we extracted four types of brain features, three functional and one structural features to discover the differences between the ASD subtypes as summarized in Figure 1A. As the first functional feature, we presented a brain pattern extraction method to determine the brain patterns and related sub-networks of different ASD subtypes. For the other two functional features, we extracted two common features, amplitude of low-frequency fluctuation (ALFF) and fractional ALFF (fALFF). Finally, for the structural feature, we extracted gray matter volume (GMV) to examine whether there are structural changes between the subtypes. In this section, we first introduce the fMRI dataset used in this study (Section 2.1). Then, we introduce the fundamental features used in this study (Section 2.2), including functional connectivity (FC), ALFF, fALFF, and GMV. As shown in Figure 1B, we proposed a tensor-decomposition-based brain pattern feature extraction method for the ASD subtypes to show brain community features (Section 2.3). Finally, we use a statistical test to check whether there are significant differences between the ASD subtypes in terms of functional and structural features (Section 2.4).

### 2.1 Data acquisition

The fMRI dataset used in this study was obtained from the Autism Brain Imaging Data Exchange I (ABIDE I) (Craddock et al., 2013; Di Martino et al., 2014), a public dataset involving resting-state fMRI and anatomical and phenotypic datasets from 17 international sites. Full details for acquisition parameters, site-specific protocols and descriptions about the participants can be found at https://fcon_1000.projects.nitrc.org/indi/abide/abide_I.html. The entire dataset contains data from 539 patients with ASD and 573 typical controls. However, since our work focused on the differences between ASD subtypes, only resting-state fMRI data and anatomical data from patients with ASD subtypes were considered for extracting functional and structural features, respectively. The inclusion criteria were as follows.

- With exact subtype label
- Without data error
- Without long-time fixed signal

The first two inclusion criteria were based on phenotypic data from the ABIDE I dataset. In terms of the third one, because it is based on the functional connectivity method, for the completeness of the article, the inclusion criteria will be described in Section 2.2.

The corresponding datasets used in this study are described in Table 1. The dataset contained resting-state fMRI and anatomical data from 152 patients with autism, 54 patients with Asperger’s, and 28 patients with Pervasive Developmental Disorder-Not Otherwise Specified (PDD-NOS). The labels of the subtypes are from the phenotypic data provided by the ABIDE project. The diagnosis of different subtypes and detailed information of the subjects are provided in the Supplementary material. To make our results more robust, we used the same preprocessed data from the ABIDE Preprocessed project (Craddock et al., 2013) with the pipeline provided by the Connectome Computation System (CCS) (Xu et al., 2015). The steps and parameters of the CCS pipeline are presented in Table 2. In this study, the fMRI data were collected using the filt_global preprocessing strategy with band-pass filtering (0.01–0.1 Hz) and global signal regression. Registration from the original to Montreal Neurological Institute’s 152 (MNI152) brain template was calculated using a combination of linear and non-linear transforms.

**Table 1:** Details of fMRI data from ABIDE I used in this study.

Site	Autism	Asperger’s	PDD-NOS	Sex/(M/F)	TR/ms	Scan Time/s	Scan Machine	Eye Status/(Open/Closed)
NYU	52	18	5	65/10	2000	360	SIEMENS 3 T Allegra	65/10
SDSU	3	7	2	11/1	2000	370	GE 3 T MR750	12/0
SBL	1	4	6	11/0	2,200	448	Philips Intera 3 T	0/11
UM1	35	6	1	36/6	2000	600	GE 3 T Signa	42/0
UM2	10	3	0	12/1	2000	600	GE 3 T Signa	13/0
USM	44	0	1	45/0	2000	486	SIEMENS TrioTim	45/0
Max_Mun	2	8	0	10/0	3,000	606	SIEMENS Verio	7/3
YALE	5	8	13	19/7	2000	400	SIEMENS TrioTim	26/0
Total	152	54	28	209/25	n.a.	n.a.	n.a.	210/24

M, male; F, female.

**Table 2:** The steps and parameters in the CCS pipeline.

Section	Step	Strategy
Basic processing	Drop first “N” volumes	4
	Slice timing correction	Yes
	Motion realignment	Yes
	Intensity normalization	4D Global mean = 1,000
Nuisance signal removal	Motion	24-param
	Tissue signals	Mean WM and CSF signals
	Motion realignment	Yes
	Low-frequency drifts	Linear and quadratic trends

### 2.2 Feature extraction

#### 2.2.1 Brain atlas

Based on the anatomical MRI and resting-state fMRI analysis, regions of interest (ROI) based features and brain networks features are extracted and analyzed to provide some significant information for the ASD subtypes discrimination. The brain data ROIs, which are defined by brain atlases, represent the averages of the values from the brain regions (voxels) with similar functions. Brain networks are even bigger concepts representing sets of different ROIs that work together to achieve higher-level cognitive functions. In this study, Craddock 200 brain parcellation (Craddock et al., 2012) was selected to obtain the ROI-based features for its data-driven nature. The Craddock 200 atlas is a gray matter mask that contains 200 ROIs, which can provide sufficient regions to discover the differences in functional and structural features. The robust performance of the atlas has also been verified in several previous studies (Liang and Xu, 2022; Kunda et al., 2023).

In terms of large-scale brain networks, this study performed an analysis on the 12 well-defined brain sub-networks provided by Power et al. (2011). To incorporate these sub-networks into the Craddock 200 atlas, we chose the adopted sub-networks provided by Ingalhalikar et al. (2021). The original networks were assigned to the Craddock 200 atlas after matching each ROI from the Craddock 200 atlas with the ROI from the Power264 atlas, based on the minimum Euclidean distance.

#### 2.2.2 Functional connectivity

Functional connectivity (FC) is an effective method for evaluating the relationships between different brain regions. FC is a common fMRI feature that numerous studies have used to identify the unique characteristics of the disease. According to the length of the time duration that is considered, FC can be divided into static and dynamic FC. Static FC evaluates the relationships between ROIs over the entire time length, while dynamic FC evaluates such relationships based on multiple time windows. Previous studies have shown that FC may have different patterns during the scan period in resting-state fMRI data from patients with ASD (Yao et al., 2016; Aggarwal and Gupta, 2019). Therefore, in this study, the dynamic FCs of each patient were extracted to evaluate the various patterns of the subtypes.

Dynamic FC was calculated based on the ROI signals in the sliding windows. Denote the time length of the sliding window is L , the TR of site i is $TR_{i}$ , then the signal length $N_{i}$ for the time window can be defined as Equation 1:


(1) $$N_{i}=\left[\frac{L}{TR_{i}}\right]$$

The length of the time window L is not chosen subjectively either. Leonardi and Van De Ville found that the length selection of the sliding window is related to the low-end cutoff frequency of the bandpass filter used in data processing (Leonardi and Van De Ville, 2015):


(2) $$L=\frac{1}{f_{\mathrm{low}}}$$

$f_{\mathrm{low}}$ in Equation 2 is the low-end cutoff frequency of the bandpass filter. In this study, the bandpass filter used in the preprocessing was 0.01 ~ 0.1 Hz, thus making the length of the time window L 100 s.

After determining the length of the sliding window, the FC in each time window can be described as a matrix of Pearson correlation coefficients between each two ROIs:


(3) $$\rho_{X,Y}=\frac{cov\left(X,Y\right)}{\sigma_{X}\sigma_{Y}}=\frac{E\left(\left(X-\mu_{X}\right)\left(Y-\mu_{Y}\right)\right)}{\sigma_{X}\sigma_{Y}}$$

$\rho_{X,Y}$ is the Pearson correlation coefficient between ROI X and ROI Y in the time window. μ is the mean value of the ROI signal in the time window and σ is the standard deviation of the ROI signal in the time window. The range of $\rho_{X,Y}$ is $-1\leq \rho_{X,Y}\leq 1$ , which represents the relationship between the two ROIs in the time window.

In addition to evaluating the relationship between ROIs, dynamic FC can also be used to check the quality of the data. As described in Section 2.1, dynamic FC was also used for the automatic selection of resting-state fMRI data. In the calculation of the dynamic FC, we sometimes found errors in the results. From Equation 3, we know that there will be an error in the calculation only when the standard deviation of the ROI signal is zero. In other words, when errors were found, at least one of the ROI signals remained unchanged during the time window. In fact, when we rechecked the original time series data, we found that some of the patient data had constant ROI signals. Therefore, in this study, dynamic FC was also used for the quality control of the data. When there was an error in the calculation, we excluded the data of the corresponding patients from the dataset (data from 15 subjects were excluded).

Finally, after calculating the dynamic FC, the FCs from patients with different ASD subtypes were grouped. However, as can be seen from Table 1, different sites had different scan times. Therefore, to facilitate subsequent processing, the data length of the dynamic FC was set to 120.

#### 2.2.3 Frequency domain features

In addition to the dynamic FC extracted from the ROI signal, which is a temporal feature based on resting-state fMRI data, we also utilized spectral features to determine the differences between ASD subtypes. In previous studies, low-frequency fluctuations in resting-state fMRI data have been proven to effectively reflect the brain activity of a subject during the scanning process (Zang et al., 2007; Hu et al., 2014). Therefore, the spectral features, ALFF and fALFF, were extracted to investigate the differences of the ROIs in each subtype.

ALFF aims to directly examine the low-frequency of each voxel. The ALFF value is defined as the average amplitude from each frequency point in the low-frequency band. To make it clear, for the time signal of voxel x , the signal $y\left(t\right)$ after bandpass-filtering can be described as Equation 4:


(4) $$y\left(t\right)=\overset{+\infty }{\underset{0}{\int }}x\left(\tau \right)\cdot h\left(t-\tau \right)d\tau$$

where $h\left(t\right)$ denotes the bandpass filter. Note the power spectrum of $y\left(t\right)$ as $Y\left(f\right)$ , then the ALFF value of voxel x can be described as Equation 5:


(5) $$\mathrm{ALFF}\left(x\right)=\frac{\int_{f_{\mathrm{low}}}^{f_{\mathrm{high}}}\sqrt{Y\left(f\right)}df}{f_{\mathrm{high}}-f_{\mathrm{low}}}$$

where $f_{\mathrm{high}}$ is the high-end cutoff frequency, and $f_{\mathrm{low}}$ is the low-end cutoff frequency. To be consistent with the ALFF features used in previous studies, the ALFF map used in this work is also from the CCS pipeline in the ABIDE Preprocessed project. Before extracting the ROI-level ALFF features, we normalized the ALFF map of each patient using the z-score method. Finally, the average of the ALFF values from the voxels belonging to the same ROI were calculated.

fALFF is also a common spectral feature in fMRI studies. In contrast to ALFF, fALFF also considers the signal of the non-filtered data. For the time signal of voxel x , note $X_{\mathrm{filtered}}\left(f\right)$ as the power spectrum after bandpass filtering and $X_{non-\mathrm{filtered}}\left(f\right)$ as the power spectrum of non-filtered data. The fALFF value of voxel x can be described as Equation 6:


(6) $$\mathrm{fALFF}\left(x\right)=\frac{\int_{f_{\mathrm{low}}}^{f_{\mathrm{high}}}\sqrt{X_{\mathrm{filtered}}\left(f\right)}df}{\int_{0}^{+\infty }\sqrt{X_{non-\mathrm{filtered}}\left(f\right)}df}$$

where $f_{\mathrm{high}}$ is the high-end cutoff frequency, and $f_{\mathrm{low}}$ is the low-end cutoff frequency. Similar to the condition in ALFF, the fALFF map is also from the CCS pipeline in the ABIDE Preprocessed project. The same normalization and ROI-level feature extraction were implemented for the fALFF extraction.

#### 2.2.4 Gray matter volume

As pointed out in previous studies, complicated functional changes or impairments occur in patients with ASD (Haghighat et al., 2021; Yang et al., 2023). Whether there are corresponding structural changes in the brain lobes or gyri remains a hot topic in research. In our study, GMV was extracted as a representation of structural features to determine whether changes in gray matter were similar to those in brain function. As mentioned in Section 2.1, the original MRI images used in this study were obtained from ABIDE anatomical data. To obtain the volume of each ROI, we used the Computational Anatomy Toolbox (CAT12) (Gaser et al., 2024) of the Statistical Parametric Mapping (SPM12) software (Ashburner, 2012) to perform the gray matter segmentation and ROI-level GMV estimation. In addition, *z*-score normalization was also adopted to compare the GMV of each ROI. To maintain consistency with the comparison above, the Craddock 200 atlas was used in the GMV calculation.

### 2.3 FC-based brain pattern extraction

Tensor decomposition is an effective method for separating attribute information from high-dimensional data. For the dynamic FC data in this study, which are composed of information on subtype, patient, ROI, and time, it is appropriate to use the tensor decomposition method to extract the brain patterns for each subtype. Previous studies have proven the effectiveness of tensor decomposition in brain data analysis (Becker et al., 2014; Aggarwal and Gupta, 2019; Zheng et al., 2021). Aggarwal et al. proposed an overlapping network identification method for multivariate vector regression-based connectivity (MVRC) based on tensor decomposition to determine the differences between patients with ASD and typical controls (Aggarwal and Gupta, 2019). However, the calculation of MVRC is complex, and the selection of the regularization parameters is empirical. In this study, we chose to use dynamic FC to perform brain pattern extraction for simplicity and objectivity. The framework for the brain pattern extraction is shown in Figure 1B.

#### 2.3.1 Group-level dynamic FC

As shown in Figure 1B, before extracting the brain patterns, a group-level dynamic FC for each ASD subtype should be produced to represent the overall characteristics of the dynamic FC within the same subtype. Generally, such group-level FC is generated by simply averaging all FC matrices across patients, which is common and easy to realize. However, simply averaging assumes that all the patients’ data have the same contribution to the construction of group-level FC, which may introduce additional noise and ignore the heterogeneity among the patients of the same subtype. To address this issue, Tucker decomposition (Tucker, 1966) was implemented to calculate the weights for each patient’s data and construct a group-level dynamic FC (Figure 1B).

For the dataset of a single ASD subtype, the FC matrices of the subjects in each time window can form a three-order tensor. In this study, the group-level dynamic FC was calculated based on these tensors. Note the three-order tensor for one time window as $T\in {\mathbb{R}}^{N\times N\times S}$ , where N is the number of ROIs, and S is the number of subjects. To obtain the group-level FC for each time point, Tucker decomposition was used to calculate the weight of the FC for each subject. The decomposition can be described as Equation 7:


(7) $$T\approx K\times_{1}X\times_{2}Y\times_{3}Z$$

where K is the core tensor of the decomposition. Because we do not need to compress or approximate the FC data, the dimensions of the core tensor can be set to be the same as those of T , i.e., $K\in {\mathbb{R}}^{N\times N\times S}$ . Besides, $X\in {\mathbb{R}}^{N\times N}$ , $Y\in {\mathbb{R}}^{N\times N}$ , $Z\in {\mathbb{R}}^{S\times S}$ are the orthonormal factor matrices along each mode of the tensor T , which contain the information of the corresponding attribute. And $\times_{n}$ is the mode n product in the decomposition. Obviously, only the matrix Z contains the information of the subjects, and from the principle of Tucker decomposition, the first column of Z contains the most significant FC features across the subjects. Therefore, in this study, $Z_{1}\in {\mathbb{R}}^{S\times 1}$ as the first column of Z , is used as the weight to construct the group-level FC:


(8) $$\bar{G}=\frac{\sum_{s=1}^{S}Z_{1}\left(s\right)M\left(s\right)}{\sum_{s=1}^{S}Z_{1}\left(s\right)}$$

In Equation 8, $\bar{G}\in {\mathbb{R}}^{N\times N}$ is the group-level FC for the time point, and M is the FC matrix of the subject. A group-level dynamic FC tensor $G\in {\mathbb{R}}^{N\times N\times W}$ can be constructed by calculating group-level FC at each time point, where W is the number of time windows.

#### 2.3.2 Extraction of brain patterns

Brain patterns are sets of brain regions that can provide important community information during brain activity. The investigation of brain patterns may further illustrate the impairment in brain function based on FC. Previous studies have shown that non-negative tensor factorization has a good ability to extract pattern information from brain data (Ponce-Alvarez et al., 2015; Aggarwal and Gupta, 2019). In this study, we performed non-negative CANDECOMP/PARAFAC (CP) decomposition with adaptation of the block principal pivoting algorithm (Kim and Park, 2008) to extract multiple brain patterns of the subtypes.

Because we are performing non-negative CP decomposition, the absolute values of all group-level dynamic FCs of the subtypes are obtained. For convenience, the following group-level dynamic FCs refer to the absolute values. Given a fixed rank R , for the group-level FC tensor $G\in {\mathbb{R}}^{N\times N\times W}$ , the decomposition of brain patterns can be described as Equation 9:


(9) $$G\approx \overset{R}{\underset{i=1}{\sum }}\lambda_{i}x_{i}\circ y_{i}\circ z_{i}$$

where “ ∘ ” is the sign of outer product, $x_{i}\in {\mathbb{R}}^{N\times 1}$ , $y_{i}\in {\mathbb{R}}^{N\times 1}$ and $z_{i}\in {\mathbb{R}}^{W\times 1}$ are the factor vectors with the column norm of 1, which contains the information of the dimensions or modes of the tensor G . $\lambda_{i}\in \mathbb{R}$ are the weights of the rank-one tensors which are produces by the outer product of $x_{i}$ , $y_{i}$ and $z_{i}$ . From the process of CP decomposition, we can know that $x_{i}$ and $y_{i}$ represent the information of the ROIs and the vector $z_{i}$ represents the information hidden in the time. Since the FCs are symmetric, the vectors $x_{i}$ and $y_{i}$ should be identical. Then, if we consider each rank-one tensor as a brain pattern, the rank R is actually the number of brain patterns hidden in the group-level FC tensor and $x_{i}$ or $y_{i}$ is actually the strength of the ROI in the corresponding pattern. Therefore, to determine the ROI communities in each brain pattern, we used the mean plus one standard deviation as the threshold value to detect ROIs with a high activity strength in the pattern.

#### 2.3.3 Determination of the number of brain patterns

From the CP decomposition introduced in Section 2.3.2, we can know that rank R is actually the number of rank-one tensors. In other words, rank R determines the number of brain patterns in this study. However, the prediction of rank R in CP decomposition is an NP-hard problem. Various studies have proposed methods for determining the appropriate R for CP decomposition. In this study, we introduced the core-consistency method (Gauvin et al., 2014) for the prediction of rank R for its simplicity and efficiency.

For Tucker decomposition, the result is composed of factor matrices related to the corresponding dimensions of the tensor. Therefore, if the process is rewritten in the form of vectors, Tucker decomposition can be expressed as Equation 10:


(10) $$T\approx \overset{I_{X}}{\underset{i_{X}=1}{\sum }}\overset{I_{Y}}{\underset{i_{Y}=1}{\sum }}\overset{I_{Z}}{\underset{i_{Z}=1}{\sum }}k_{i_{X}i_{Y}i_{Z}}x_{i_{X}}\circ y_{i_{Y}}\circ z_{i_{Z}}$$

where $x_{i_{X}}$ , $y_{i_{Y}}$ , and $z_{i_{Z}}$ are the columns of the original factor matrices X , Y and Z , respectively. $k_{i_{X}i_{Y}i_{Z}}$ is the element of the kernel tensor K . Comparing (9) and (10), we can find that when the kernel tensor K is a superdiagonal tensor, Tucker decomposition degenerates into CP decomposition. This implies that CP decomposition is a special form of Tucker decomposition. Then, for the fixed vectors $x_{i}$ , $y_{i}$ and $z_{i}$ of the CP decomposition (without the constraint that column is 1), with an appropriate rank R, the core tensor in Tucker decomposition should be similar to the superdiagonal tensor of ones. Thus, core consistency can be calculated as Equation 11:


(11) $$\mathrm{core}\;\mathrm{consistency}=100\times \left(1-\frac{\sum_{i=1}^{R}\sum_{j=1}^{R}\sum_{k=1}^{R}{\left(k_{ijk}-1\right)}^{2}}{R}\right)$$

where $k_{ijk}$ is the element in the Tucker decomposition. In this study, we calculated the core-consistency value from R=1 to R=10 to find the appropriate R value, and to ensure the reproducibility of the results, 3-fold cross-validation method was implemented. The results are shown in Figure 1C. As shown in Figure 1C, the core consistency values of the three ASD subtypes varied from *R* = 1 to *R* = 10, and we found a rapid decrease from *R* = 2 to *R* = 7. The research of Rasmus Bro et al. found that a core consistency value in the neighborhood of 50% leads to a problematic model (Bro and Kiers, 2003). Therefore, in this study, to find as many brain patterns as possible while ensuring the stability of the result, 60% is considered as an appropriate threshold, and the corresponding R values with the core consistency above the threshold are considered as the optimal number of brain patterns for each ASD subtype. As a result, the R values for autism were 3, and for Asperger’s and PDD-NOS were 4.

### 2.4 Statistical test

In this study, in addition to the comparison of brain patterns, we aimed to determine the differences between ASD subtypes by inter-comparison. To achieve this goal, the functional and structural feature values including ALFF, fALFF, and GMV of each ROI from different subtypes were compared by t-test to determine the differences between the subtypes. Furthermore, to discover such differences on a larger scale, we used the 12 well-defined brain networks parcellation to check the sub-networks that were involved in the comparisons. Because these comparisons involve multiple testing, in this work, the false discovery rate (FDR) correction was used for multiple testing correction. In addition, to check which ROIs were involved in all these comparisons (brain pattern, ALFF, fALFF, and GMV), we compared all the comparisons results and selected the overlapping ROIs. It should be noted here that for the comparison of brain patterns of the subtypes, a ROI was counted as involved in the comparison of one brain pattern if it appeared in one subtype and did not appear in the other.

## 3 Results

In this section, we present the results of the tensor decomposition-based brain pattern extraction and compare the functional and structural features. The brain patterns of each ASD subtype are described in Section 3.1. The inter-comparisons of ALFF, fALFF, and GMV between the subtypes are shown in Section 3.2. It should be noted that all the results from the comparisons based on the t-test in this study were at a significance level of 0.05, and FDR correction was adopted for multiple testing correction.

### 3.1 Brain pattern extraction

**Figure 2:**
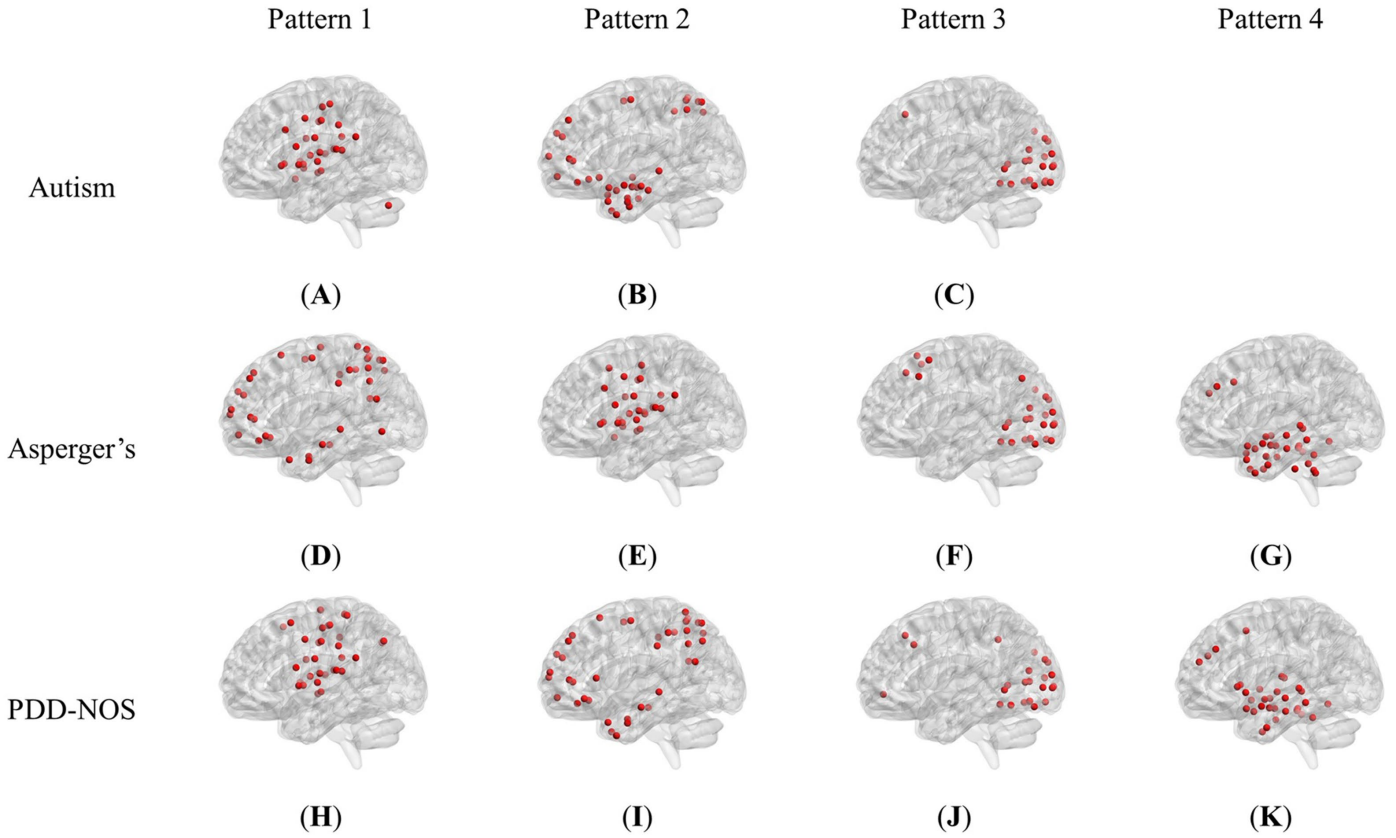
Brain patterns of the ASD subtypes: **(A)** Pattern 1 of autism (dominated by AN and CON). **(B)** Pattern 2 of autism (dominated by DMN and DAN). **(C)** Pattern 3 of autism (dominated by VN). **(D)** Pattern 1 of Asperger’s (dominated by DMN and DAN). **(E)** Pattern 2 of Asperger’s (dominated by AN and CON). **(F)** Pattern 3 of Asperger’s (dominated by VN). **(G)** Pattern 4 of Asperger’s (dominated by SCN and DMN). **(H)** Pattern 1 of PDD-NOS (dominated by AN, SMH and CON). **(I)** Pattern 2 of PDD-NOS (dominated by DMN and DAN). **(J)** Pattern 3 of PDD-NOS (dominated by VN). **(K)** Pattern 4 of PDD-NOS (dominated by SCN and DMN). AN, auditory network; CON, cingulo-opercular network; SCN, subcortical network; SMM, sensory/somatomotor mouth; SMH, sensory/somatomotor hand; SAN, salience network; FPN, fronto-parietal network; DMN, default mode network; DAN, dorsal attention network; VN, visual network; VAN, ventral attention network; CPN, cingulo-parietal network.

Figure 2 shows the brain patterns of each ASD subtype. Based on the results of the core consistency analysis as shown in Figure 1C, the number of brain patterns differed depending on subtypes. The patient group with autism tended to have three different brain patterns, while the patient groups with Asperger’s and PDD-NOS tended to have four different brain patterns. To clearly show the spatial distribution of the ROIs of the brain patterns, we projected the nodes onto a standard brain template using the BrainNet Viewer toolbox (Xia et al., 2013). As shown in Figure 2, the brain patterns extracted by the tensor decomposition method show a high degree of organization.

To demonstrate how these functional networks vary among subtypes, we followed the strategy adopted by Ingalhalikar et al. (2021) to divide the ROIs into 12 well-defined sub-networks based on different brain functions, including sensory/somatomotor hand (SMH), sensory/somatomotor mouth (SMM), cingulo-opercular network (CON), auditory network (AN), default mode network (DMN), cingulo-parietal network (CPN), visual network (VN), fronto-parietal network (FPN), salience network (SAN), subcortical network (SCN), ventral attention network (VAN), and dorsal attention network (DAN). Table 3 shows the sub-networks involved in different brain patterns and the number of ROIs that belong to the sub-networks (sub-networks with only one ROI are not listed). From Table 3, we can see that if we consider the sub-networks with more than five ROIs as the dominant sub-network in the brain pattern, we can find that some of the brain patterns of different subtypes can be grouped together. Pattern 1 of autism, Pattern 2 of Asperger’s and Pattern 1 of PDD-NOS can be gathered as Group 1, Pattern 2 of autism, Pattern 1 of Asperger’s and Pattern 2 of PDD-NOS can be gathered as Group 2, Pattern 3 of the three subtypes can be gathered as Group 3, and finally Pattern 4 of Asperger’s and PDD-NOS can be gathered as Group 4. We found that the sub-networks of Group 1 were mainly AN and CON, which were related to auditory activity, tonic alertness, and task control, while Pattern 1 of PDD-NOS showed a difference in SMH, which is linked to the motor activity of the hands. Group 2 mainly included the ROIs from the DMN and DAN, which are related to resting-state activity and task attention. Previous studies have pointed out that the activity of areas from the DMN is anti-correlated with that of the DAN regions (Wang J. et al., 2019; Qian et al., 2020). In this study, since we analyzed the brain patterns extracted by the non-negative CP decomposition, these two sub-networks were gathered in the same pattern, which is consistent with previous studies. Group 3 is mainly correlated with the VN, which is from Pattern 3 of all subtypes. Finally, Group 4 only included the brain patterns of Asperger’s and PDD-NOS, which are the patterns containing the ROIs from the SCN and DMN. Abnormalities in the FC of the SCN and DMN may lead to the main difference between autism and the other subtypes.

**Table 3:** Sub-networks involved in brain patterns.

ASD subtype	Brain pattern	Sub-network (number of ROIs)
Autism	1	**AN(9), CON(7)**, SCN(3), SMM(3), SMH(2), SAN(2), FPN(2)
	2	**DMN(20), DAN(5)**, SCN(3), FPN(2)
	3	**VN(15)**, VAN(2)
Asperger’s	1	**DMN(19), DAN(7)**, SMH(4), VAN(2), FPN(2)
	2	**AN(8), CON(8)**, SCN(4), SMM(3), SAN(2)
	3	**VN(15)**, DMN(3), FPN(3), VAN(2)
	4	**SCN(13), DMN(10)**, SMH(2)
PDD-NOS	1	**AN(9), SMH(6), CON(6)**, SMM(3), FPN(3), SCN(2), DMN(2)
	2	**DMN(22), DAN(6)**, VAN(3), FPN(2), SMH(2)
	3	**VN(15)**, FPN(3), VAN(2)
	4	**SCN(14), DMN(5)**, SAN(4), CON(2), SMH(2)

AN, auditory network; CON, cingulo-opercular network; SCN, subcortical network; SMM, sensory/somatomotor mouth; SMH, sensory/somatomotor hand; SAN, salience network; FPN, fronto-parietal network; DMN, default mode network; DAN, dorsal attention network; VN, visual network; VAN, ventral attention network; CPN, cingulo-parietal network. Sub-networks with more than five nodes (include five) are shown in bold.

### 3.2 Inter-comparison of the ASD subtypes

**Figure 3:**
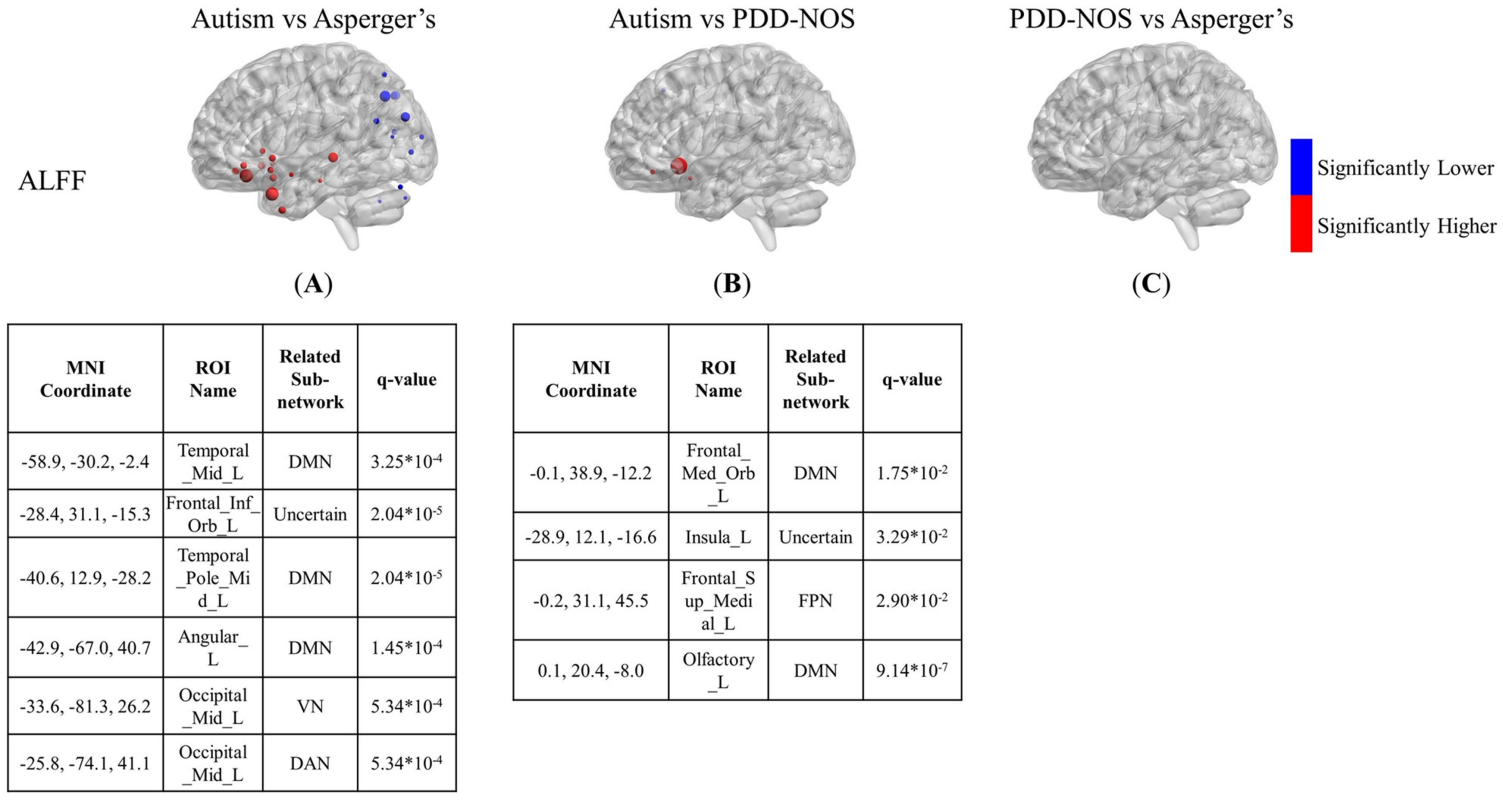
The results of the inter-comparison for ALFF: **(A)** is the result of the comparison between autism and Asperger’s; **(B)** is the result of the comparison between autism and PDD-NOS; **(C)** is the result of the comparison between PDD-NOS and Asperger’s. The tables under each subgraph show the ROIs with the top five of the smallest *q*-values (if there are not enough ROIs, then all surviving ROIs are shown). It should be noted that the *q*-values in this study are *p*-values after FDR correction. The sizes of the ROIs in the figure are related to their *q*-values. A larger ROI node indicated a smaller *q*-value, showing greater significance in the *t*-test. AN, auditory network; CON, cingulo-opercular network; SCN, subcortical network; SMM, sensory/somatomotor mouth; SMH, sensory/somatomotor hand; SAN, salience network; FPN, fronto-parietal network; DMN, default mode network; DAN, dorsal attention network; VN, visual network; VAN, ventral attention network; CPN, cingulo-parietal network.

**Figure 4:**
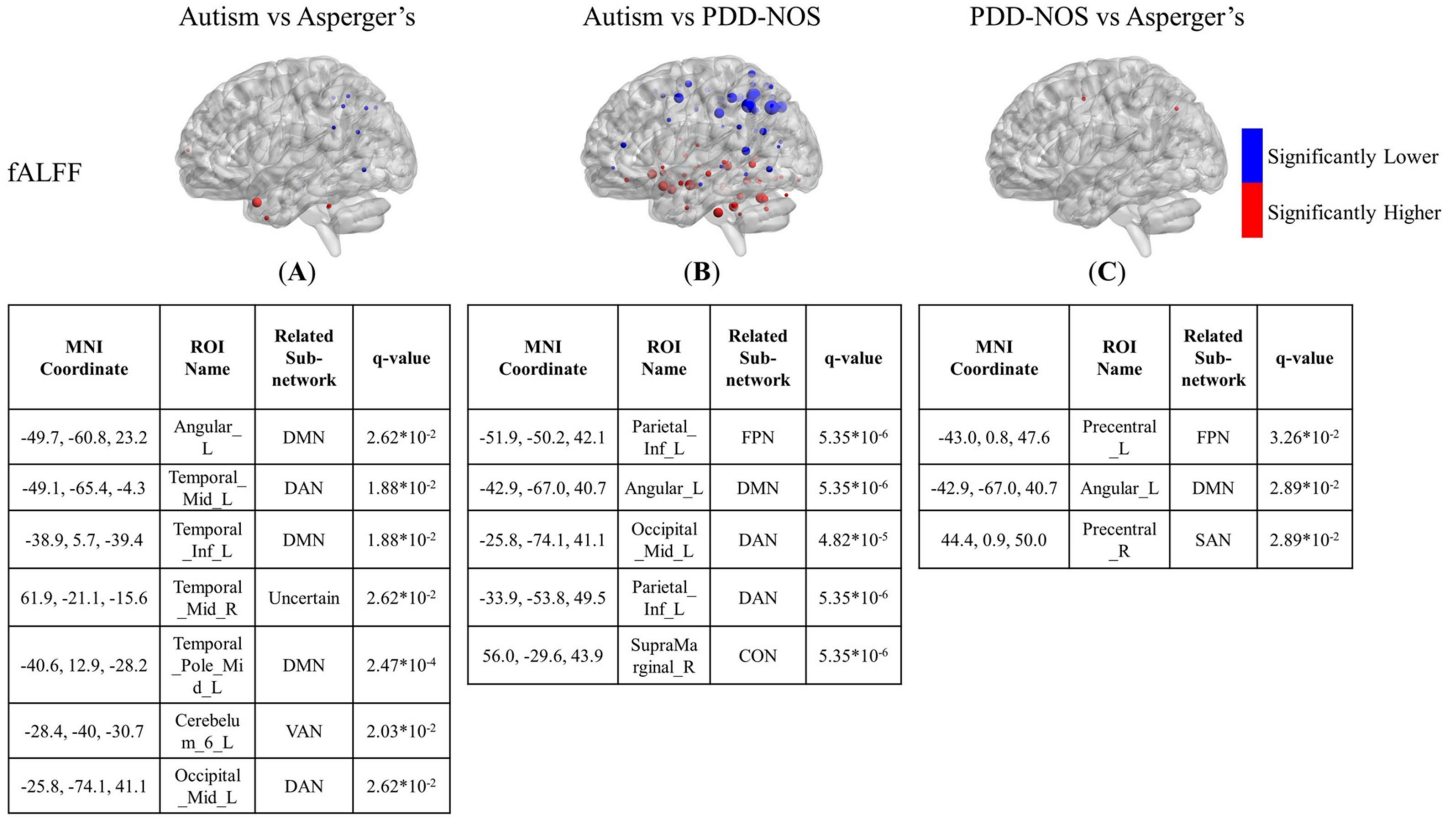
The results of the inter-comparison for fALFF: **(A)** is the result of the comparison between autism and Asperger’s; **(B)** is the result of the comparison between autism and PDD-NOS; **(C)** is the result of the comparison between PDD-NOS and Asperger’s. The tables under each subgraph show the ROIs with the top five smallest *q*-values. AN, auditory network; CON, cingulo-opercular network; SCN, subcortical network; SMM, sensory/somatomotor mouth; SMH, sensory/somatomotor hand; SAN, salience network; FPN, fronto-parietal network; DMN, default mode network; DAN, dorsal attention network; VN, visual network; VAN, ventral attention network; CPN, cingulo-parietal network.

**Figure 5:**
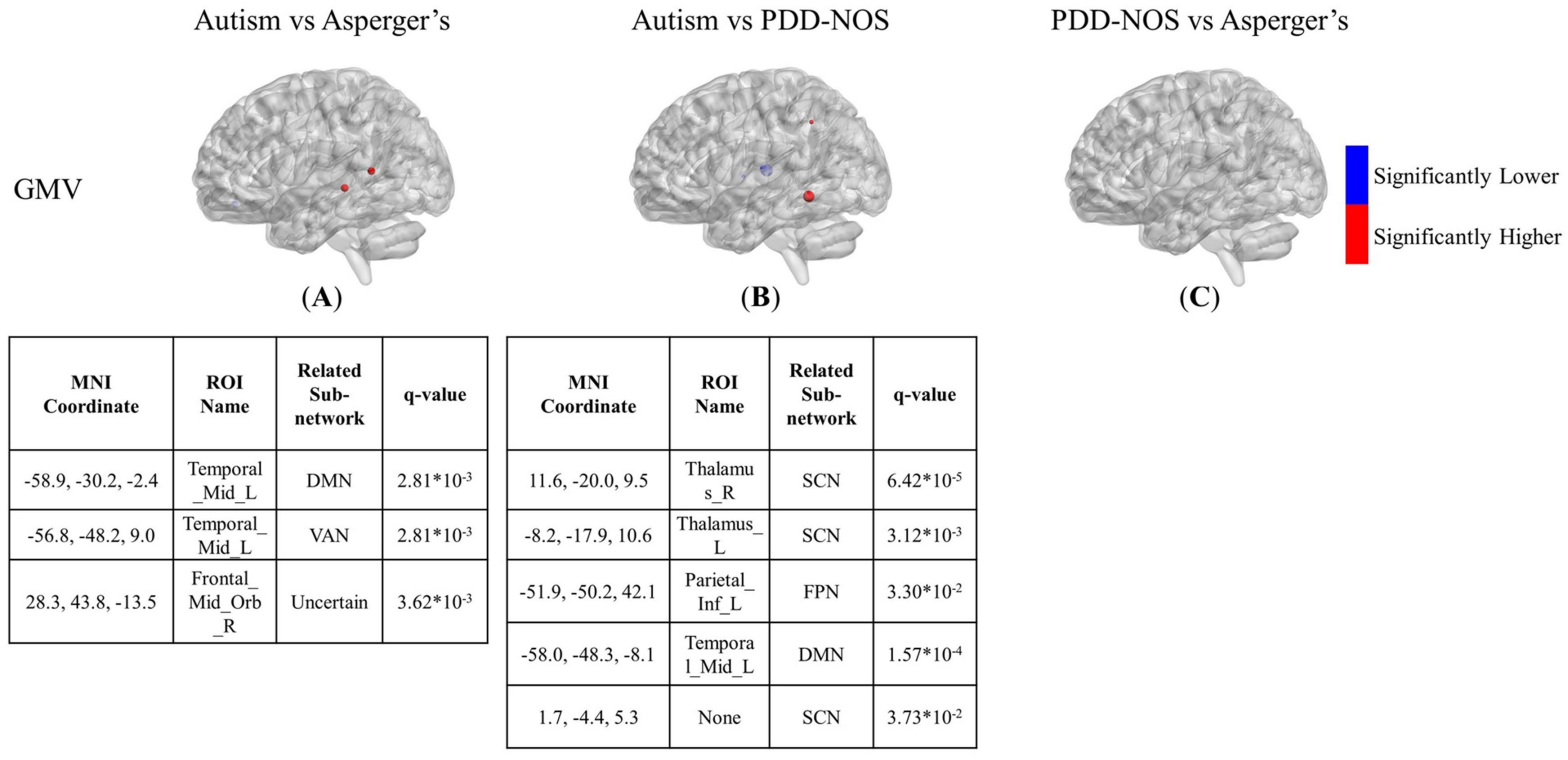
The results of the inter-comparison for GMV: **(A)** is the result of the comparison between autism and Asperger’s; **(B)** is the result of the comparison between autism and PDD-NOS; **(C)** is the result of the comparison between PDD-NOS and Asperger’s. The tables under each subgraph show the ROIs with top 5 of the smallest *q*-value. AN, auditory network; CON, cingulo-opercular network; SCN, subcortical network; SMM, sensory/somatomotor mouth; SMH, sensory/somatomotor hand; SAN, salience network; FPN, fronto-parietal network; DMN, default mode network; DAN, dorsal attention network; VN, visual network; VAN, ventral attention network; CPN, cingulo-parietal network.

Figures 3–5 show the results of the inter-comparison of the ASD subtypes for ALFF, fALFF, and GMV, respectively. In terms of ALFF, as shown in Figure 3, the number of different ROIs between autism and Asperger’s was much greater than that in the other two conditions, with 27 significant nodes. However, there was no ROI shown in the comparison of PDD-NOS and Asperger’s, indicating that the difference between PDD-NOS and Asperger’s in ALFF was not significant at the level of 0.05. The differences between autism and Asperger’s are mainly focused on the ROIs from the DMN, SCN, VN, and some scattered ROIs from the SAN, CON, DAN, CPN, VAN, and FPN (only one ROI for each). The ROIs with significantly higher ALFF values were mainly in the frontal and temporal lobes, whereas the ROIs with significantly lower ALFF values were mainly in the parietal and occipital lobes. The significant ROIs in the autism vs. PDD-NOS comparison are much fewer, with only four nodes from DMN and FPN, which are mainly focused on the frontal lobe and insula cortex.

In terms of fALFF, the situation is the opposite to that of ALFF. The differences between autism and Asperger’s in the fALFF are not as many as in the ALFF. As shown in Figure 4A, the significantly different ROIs were mainly focused on the DMN, DAN, VAN, and FPN, with scattered ROIs from the SCN and CON. The DMN still showed the most different ROIs in the comparison, while ROIs from the DAN and FPN of the autism group showed significantly lower fALFF values than those of the Asperger’s group. The comparison between autism and PDD-NOS showed significantly more significant nodes than the other two conditions, with 86 ROIs appearing to be significantly different between the two subtypes. Unlike the condition of autism vs. Asperger’s, the SCN from the autism group showed the most different nodes compared with the PDD-NOS group, where 19 ROIs showed significantly higher fALFF values. Nodes from the DMN, DAN, VAN, and FPN also appear in the comparison. In addition, nodes from VN and CON show differences that are not found in the comparison between autism and Asperger’s condition. There were only three nodes showing differences between Asperger’s and PDD-NOS, with two of them from the precentral gyrus and one from the angular gyrus.

The differences between the three subtypes of GMV are shown in Figures 5A–C. The significant nodes are much fewer than in the ALFF and fALFF conditions. In the comparison between autism and Asperger’s, there are 3 ROIs showing different GMV values between the two subtypes, with two nodes for higher values in the middle temporal gyrus and one for lower values in the orbital part of the middle frontal gyrus. In the comparison between autism and PDD-NOS, the number of different nodes was slightly higher. Three ROIs from the SCN of the autism group showed significantly lower GMV values, while one ROI from the FPN and DMN showed higher GMV values than the PDD-NOS group. For the comparison between Asperger’s and PDD-NOS, no ROI survived after the t-test, showing no statistical difference between the two subtypes in terms of GMV.

**Figure 6:**
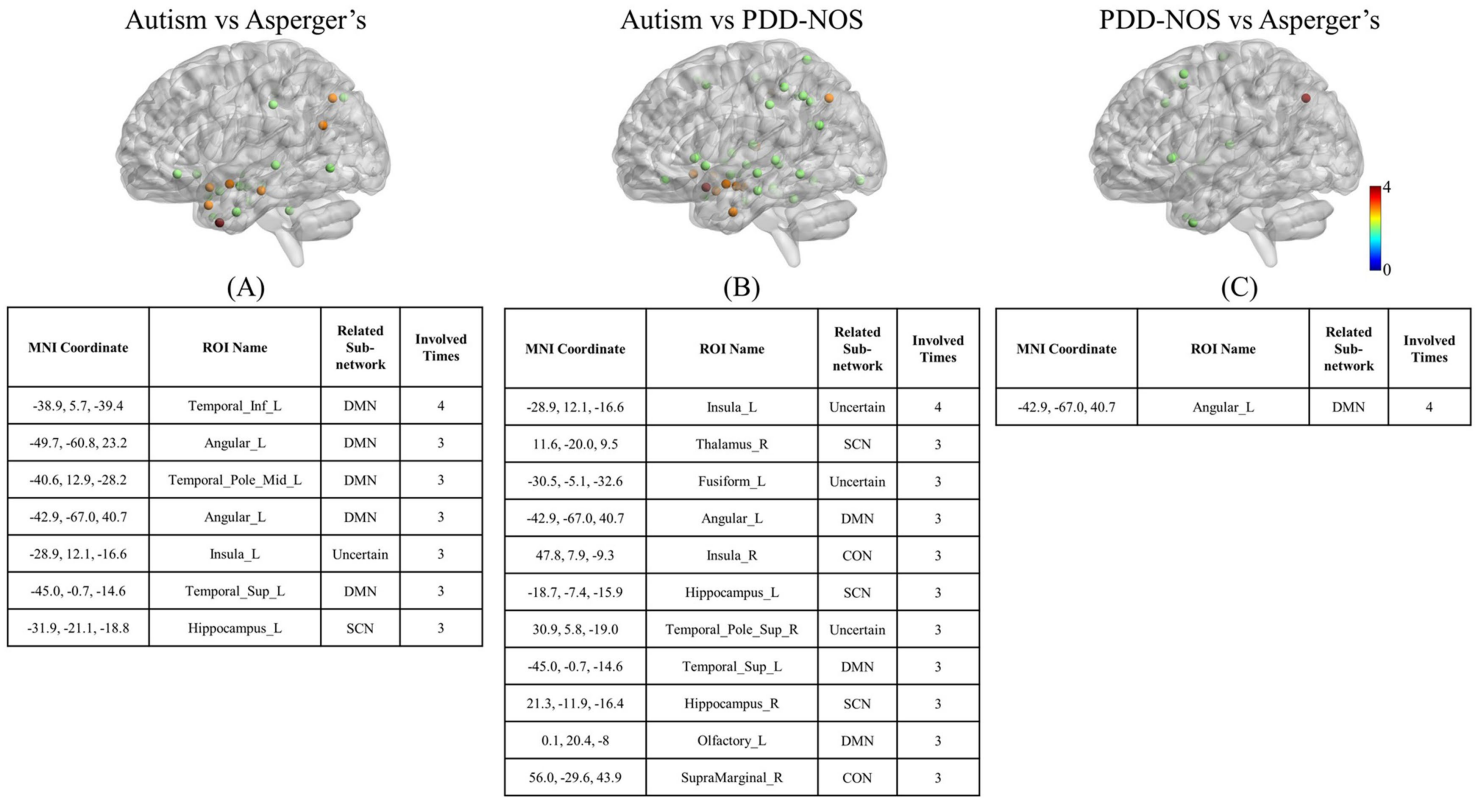
ROIs involved in multiple comparisons. **(A)** is the result of the nodes involved in the comparisons between autism and Asperger’s; **(B)** is the result of the nodes involved in the comparisons between autism and PDD-NOS; **(C)** is the result of the nodes involved in the comparisons between PDD-NOS and Asperger’s. The nodes in the figures are the ROIs involved in at least two comparisons and the nodes in the tables are the ROIs involved in more than two comparisons. AN, auditory network; CON, cingulo-opercular network; SCN, subcortical network; SMM, sensory/somatomotor mouth; SMH, sensory/somatomotor hand; SAN, salience network; FPN, fronto-parietal network; DMN, default mode network; DAN, dorsal attention network; VN, visual network; VAN, ventral attention network; CPN, cingulo-parietal network.

The ROIs involved in multiple comparisons are shown in Figure 6. Angular_L, Temporal_Sup_L, and Insula_L are the high-frequency ROIs involved in multiple comparisons in autism and the other two subtypes. Interestingly, except for Insula_L, which does not belong to a certain network, the other two are from the DMN. In addition, in the result of PDD-NOS vs. Asperger’s, Angular_L from the DMN also showed abnormalities in multiple comparisons. In terms of the other ROIs, in the comparisons between autism and Asperger’s, the abnormality in DMN and SCN remained the main difference, while in the comparison between autism and PDD-NOS, the ROIs from CON also appeared in the multiple comparisons. Since the ROIs of CON mainly show the difference in the brain pattern comparison, this result may indicate an abnormality in the functional connectivity or brain community of CON between these two subtypes. In terms of the comparisons between PDD-NOS and Asperger’s, only Angular_L from DMN showed a high frequency of involvement in the comparisons, once again showing fewer differences than the other two conditions (autism vs. Asperger’s and autism vs. PDD-NOS).

## 4 Discussion

In this study, we compared the neuroimaging features of ASD subtypes based on multiple criteria including brain patterns, ALFF, fALFF, and GMV. In the brain pattern extraction, we found abnormalities in the FC of the SCN and DMN, which may lead to the main difference between autism and the other subtypes. In addition, in the inter-comparisons, ALFF, fALFF, and GMV showed different abilities to capture the functional and structural characteristics of the subtypes. For ALFF and fALFF, the ROIs from DMN also showed different activities among the three subtypes. ROIs from SCN also showed abnormalities in the comparisons between autism and the other two subtypes, which is consistent with the results of the brain pattern extraction. In addition, multiple ROIs from DAN, VAN, and FPN appeared in the comparison of fALFF between autism and PDD-NOS. Compared to the comparison between autism and the other two subtypes, the comparison between Asperger’s and PDD-NOS showed that few ROIs were significantly different. In terms of GMV, only three ROIs showed themselves in the comparisons between autism and the other two subtypes, and no ROI showed significant differences in the comparison between Asperger’s and PDD-NOS, showing less severe effects in the brain structures of the three subtypes. To further discuss our findings, in this section, the results obtained from the patterns, ALFF, fALFF, and GMV for each ASD subtype, as described in Section 3, will be combined to analyze the characteristics of the subtypes and compared with previous studies. In Section 4.1, the results of the brain pattern extraction are discussed, and in Section 4.2, our findings on brain patterns, ALFF, fALFF, and GMV are discussed together and compared with previous studies. Finally, in Section 4.3, the limitations of the study are discussed.

### 4.1 Brain patterns analysis

Brain pattern is a method used to gather different communities of ROIs in brain activity. Multiple methods have been used to extract brain patterns. In this study, we used the tensor decomposition method to capture multiple brain patterns hidden in the resting-state fMRI scan process. As introduced in Section 2.3, by performing on the dynamic FC matrices, our brain pattern extraction method is composed of group-level dynamic FC construction and pattern decomposition. The group-level dynamic FC construction part was realized by utilizing Tucker decomposition to evaluate the activation of each individual subject, and the pattern decomposition was realized by utilizing non-negative CP decomposition to capture the different brain communities that are hidden in the group-level dynamic FC by combining with each other. As noted by Aggarwal and Gupta (Aggarwal and Gupta, 2019), such a method can be used to extract overlapping dynamic functional brain networks, which means that a single ROI can appear in multiple brain patterns. Such a characteristic can also be found in the calculation of pattern decomposition, as there is no limitation for the ROIs to be selected in different brain patterns. Compared with the method proposed in (Aggarwal and Gupta, 2019), the brain pattern extraction method proposed in this study has two obvious advantages: (1) the dynamic FC is calculated using the Pearson correlation coefficient, which avoids the influence of empirical hyperparameters on the calculation, and (2) the brain patterns are extracted with a higher threshold, which ensures the quality of decomposition and reserves the mode of sub-networks. As shown in Figure 5, the nodes of the brain patterns extracted from each ASD subtype showed a compact distribution in functional areas, with no well-defined sub-networks appearing in more than two brain patterns, which is consistent with the division of the sub-networks. Furthermore, such distributions will also make it easier to analyze the brain functions involved in brain patterns.

To the best of our knowledge, this is the first study to extract brain patterns of the three ASD subtypes. From Table 3, we can see that each brain pattern has different functions. Furthermore, although not exactly the same, there are correspondences between brain patterns in the three subtypes, which are described as groups in Section 3.1. We noticed that in Groups 1 and 2, the sequence of the brain patterns from Asperger’s was different from that of the other subtypes. This is an interesting phenomenon, which means that not only the distribution of ROIs in the community, but also the order of the brain pattern may illustrate the differences between the subtypes. In the calculation of non-negative CP decomposition, in addition to the pattern information of the dimensions, there is also a weight λ combined with the pattern. A higher-ranked brain pattern indicates a higher λ value, thus leading to higher energy in group-level dynamic FC. Therefore, although there are not many differences in the brain community in Groups 1 and 2, the abnormality of Asperger’s in Pattern 1 and Pattern 2 may also be considered as a significant characteristic of Asperger’s, compared with the other two subtypes.

In terms of the ROIs distributed in the brain patterns, as described in Section 3.1, the most significant difference in the brain patterns was the lack of Pattern 4 in autism. It should be noted that the lack of the pattern here does not mean that the correlated ROIs and sub-networks are inactive, which indicates that the energy of the community is insufficient to be recognized as a pattern in brain pattern extraction. However, this also reflects an abnormality in connectivity in the brain community. In addition, compared with the results of the inter-comparison, we also found that the differences between Asperger’s and PDD-NOS are not as many as the differences between autism and Asperger’s or PDD-NOS, showing similar results to the method proposed in this work. In terms of the analysis of the brain functions of the three subtypes, the *t*-test results of ALFF and fALFF were also somewhat similar to the brain patterns we obtained in Section 3.1. These similarities and differences are described in Section 4.3. In addition, as described in Section 3.1, Pattern 3 of all subtypes are correlated with the VN. This interesting phenomenon indicates that patients of the three subtypes may experience similar visual activity during the scan process; therefore, we checked the instructions of the resting-state fMRI scan process from each site and found that seven out of eight sites involved in this study required patients to keep their eyes open. It appears that this instruction is directly reflected in the brain patterns, which further demonstrates the effectiveness of our method.

### 4.2 Comparison of the subtypes

Autism is such a typical subtype of ASD that many studies just take “autism” as the short for ASD (Daly et al., 2012; Clery et al., 2013; Fan et al., 2014; Leming et al., 2020). ASD is defined based on the core symptoms of typical autism (Tang, 2021). The autism subtype is dominant among ASD patients, which is also evident from the proportion of the three subtypes in the ABIDE dataset used in this study. In this study, we aimed to identify biomarkers for the detection of ASD subtypes. The result of the brain patterns shows that the brain patterns of autism group can be mainly divided into three types, which are dominated by AN and CON, DMN and DAN, and VN, respectively. The lack of Pattern 4 in the autism group may suggest that the variation in the functional connectivity in the SCN and part of the DMN leads to the main difference between autism and the other two subtypes. Such differences in the DMN can also be observed in the inter-comparison of ALFF and fALFF. Abnormal activity in the DMN is considered to be the typical difference between patients with ASD and healthy controls in previous studies (Guo et al., 2019; Mash et al., 2019). From the results of the brain patterns and t-test, it can be inferred that abnormalities in the DMN can also be important biomarkers for the discrimination of ASD subtypes. As found in Yang’s research, the DMN shows different intra-and inter-module connections in the three ASD subtypes (Yang et al., 2023). It is also pointed out that the DMN has unique roles across ASD subtypes (Qi et al., 2020). In addition to the DMN, abnormalities in the SCN are also significantly different between autism and the other two subtypes. In Qi’s study, the main differences between Asperger’s, PDD-NOS, and autism were the functional subcortical brain areas (Qi et al., 2020). Our study observed a similar phenomenon in the comparison of brain patterns and the inter-comparison of ALFF and fALFF. Interestingly, such a difference in SCN did not appear simultaneously in one comparison. For example, the lack of the pattern for SCN shows the aberrant brain community of autism, while the comparisons of ALFF and fALFF show abnormal brain function between autism and the other two subtypes. From the results of our work, it can be suggested that there are differences in both brain function and region collaboration with the ROIs in the DMN and SCN between autism and the other subtypes, while the regional collaboration abnormality between Asperger’s and PDD-NOS is limited.

In addition to the brain patterns, there were unique findings in the inter-comparison. The ALFF and fALFF tests showed different abilities to capture the differences between the subtypes. ALFF was more sensitive in detecting the functional variation between autism and Asperger’s, whereas fALFF was more sensitive in determining the differences between autism and PDD-NOS. Apart from the nodes from the SCN and DMN mentioned above, we also found low VN values for the regions of autism, compared with Asperger’s. However, since the group with autism was much larger than the other groups and some of the sites asked the participants to keep their eyes closed, which may affect the distribution of the functional features in VN, whether such differences are due to the different pathologies of the subtypes still needs further analysis. In the results of the inter-comparison, we found low values of fALFF in DAN and FPN for autism compared with Asperger’s. The DAN is a brain network that includes key nodes in the bilateral intraparietal sulcus and frontal eye fields and is primarily involved in applying top-down selection for stimuli and responses (Menon and D’Esposito, 2022). Previous behavioral attention studies have pointed out that there is impairment of DAN in children with ASD compared to typically developing controls (TD) (Fan et al., 2002; Pruett et al., 2011). From the results of the inter-comparison for fALFF, such impairment can also be observed in autism compared with the other two subtypes. It can be suggested that the level of attention deficit varies among ASD subtypes. In addition, ROIs of the FPN, which is also a representative prefrontal cortex network, show a larger scale of low values compared with PDD-NOS. Similar to the condition of DAN, in a functional network study of ASD (Haghighat et al., 2021), significant connectivity changes in the middle frontal gyrus and inferior frontal gyrus, which are important parts of the FPN, have been found in the comparison between ASD and TD. Such changes in brain activity in the two gyri were also found in our study, with significantly lower values in autism than in PDD-NOS. In addition, abnormalities in the VAN were found in the comparison between autism and PDD-NOS, showing four ROIs with significantly higher fALFF values and three with significantly lower values, which may indicate different impairments in the ability to detect behaviorally relevant stimuli (Menon and D’Esposito, 2022). In terms of the comparison between Asperger’s and PDD-NOS, ALFF and fALFF show a high degree of similarity, with few differences between the two subtypes, which is also suggested in the brain pattern analysis. In our study, only three nodes from the precentral gyrus and angular gyrus were different in the fALFF. In fact, PDD-NOS itself is usually called “atypical autism,” which suggests that PDD-NOS may not be a well-defined disorder but a set of disorders failing to meet the criteria for autism or Asperger’s. Such heterogeneity makes it difficult to distinguish PDD-NOS using a definite method. As pointed out in Walker’s paper (Walker et al., 2004), no differences were observed between the PDD-NOS and Asperger’s groups in any variable measuring the level of functioning.

In the comparison of GMV, only a few ROIs were found to be significantly different between the subtypes. This indicates that the functional differences between the subtypes were not always accompanied by significant changes in GMV at the ROI level. In the comparison of autism and Asperger’s, increased GMV was found in the nodes of left middle temporal gyrus in autism, which are also important structural differences between autism and TD (Abell et al., 1999; Waiter et al., 2004; Ecker et al., 2012). As noted by Ecker’s work (Ecker et al., 2012), increases in GMV in this region are correlated with increased social symptom severity in ASD patients. Asperger’s, as an ASD subtype with less severe symptoms and more higher functioning, may be a possible reason for the smaller GMV compared with autism. In the comparison between autism and PDD-NOS, as mentioned above, increased GMV was also found in the left middle temporal gyrus in autism. In addition, three ROIs from the SCN showed decreased GMV in autism compared with PDD-NOS. Considering the abnormality of SCN in brain patterns and fALFF in autism, this may suggest evidence for the reflection of abnormalities in function and structure.

In this study, we analyzed four types of brain features, including brain pattern, ALFF, fALFF and GMV, to explore the difference between ASD subtypes based on ROI-level and sub-network-level. To the best of our knowledge, this is the first study to adopt the tensor decomposition method to extract the brain patterns of the three ASD subtypes. Generally, we explored the differences between the ASD subtypes in four brain features and the functional differences in the subcortical network and default mode network of autism were found to lead to major differences from the other two subtypes, while the structural differences were limited in GMV. These findings provide a systematic comparison of the three common ASD subtypes, and may offer potential features for distinguishing between them.

### 4.3 Limitations

In this study, all the functional and structural data were from the ABIDE dataset, which is composed of MRI and fMRI data from multiple sites. Although we have dealt with the variation in the TRs of different sites, the heterogeneity of the MRI machines from each site may still have an influence on the features, including ALFF and fALFF. To address this problem, we used z-score normalization to normalize the features before inter-comparisons. Another limitation is that eye status was not considered in our study. From one perspective, the lack of standardization may have influenced our findings in the visual network; however, from another perspective, the numbers of patients with Asperger’s and PDD-NOS are small and there is a big gap between the numbers of patients with the three subtypes, and further data screening will affect the stability of the analysis results. In fact, we tried to exclude the eye-closed data and control the site difference. However, as the results provided in the Supplementary materials, the lack of data has a more serious impact on the statistical results than the site difference and eye status, especially for the subtypes with a small data size. In our work, we used non-negative CP decomposition to capture the brain community information in the resting-state fMRI scan process, and we found valuable brain pattern differences between the subtypes; however, the way in which brain networks with antagonistic functions work still needs further analysis. Finally, although we used features including brain patterns, ALFF, fALFF, and GMV to evaluate the differences between the subtypes, topological features, including module degree and participation coefficient, can also be used to capture more information about the ASD subtypes, which may provide a more comprehensive perspective to provide a deeper understanding of ASD. Additionally, ReHo might offer potential insights in local neural synchronization, which could be considered in future studies as a complementary analysis to further investigate local connectivity differences.

## Data Availability

The original contributions presented in the study are included in the article/Supplementary material, further inquiries can be directed to the corresponding author.
