# Supplementary material for: Comparison of autism spectrum disorder subtypes based on functional and structural factors
Source: Front Neurosci. 2024 Oct 4;18:1440222. doi: 10.3389/fnins.2024.1440222 (PMC11486766; doi:10.3389/fnins.2024.1440222)
Supplement: Supplementary file 1 [file Table_1.docx]

Supplementary Material

# Diagnosis of the three ASD subtypes

- NYU

Inclusion as a participant with ASD required a clinician's DSM-IV-TR diagnosis of Autistic Disorder, Asperger's Disorder, or Pervasive Developmental Disorder Not-Otherwise-Specified, which was supported by review of available records, an Autism Diagnostic Observation Schedule1-3, review of the participant's history, and when possible, an Autism Diagnostic Interview-Revised4,5.

- SDSU

Clinical diagnoses were confirmed using the Autism Diagnostic Interview-Revised, the Autism Diagnostic Observation Schedule, and expert clinical judgment according to DSM-IV-TR criteria.

- SBL

DSM-IV-TR diagnosis of Autism Spectrum Disorders established by an experienced clinician

- UM1

Diagnosis of ASD was based on the Autism Diagnostic Interview Revised (ADI-R)^9^ and the Autism Diagnostic Observation Schedule (ADOS)^10^, as well as clinical consensus. ASD youth must have completed a valid Module 3 or 4 of the ADOS (i.e., had fluent language).

- UM2

Diagnosis of Asperger's Disorder/high functioning autism (HFA) is based on the Autism Diagnostic Interview Revised (ADI-R)^4^ and the Autism Diagnostic Observation Schedule (ADOS)^5^ and clinician's consensus. Verbal IQ must be ≥80 for participants with ASD and age must be at least 13 years old. Adolescents with ASD must have completed a valid Module 3 or 4 of the ADOS (i.e., has fluent language).

- USM

Inclusion as an individual with ASD required meeting full ADOS-G, and DSM-IV-TR criteria for autism at initial ascertainment. Two subjects were included that met criteria for autism, but on subsequent assessment in a longitudinal study were found to have scores more consistent with PDD-NOS and Asperger Syndrome, respectively. All individuals with ASD are personally evaluated by a clinical autism expert.

- Max_Mun

Adult individuals were included if they had a documented clinical diagnosis according to ICD-10 criteria (F.84.5) supported by the Autism Diagnostic Interview Revised (ADI-R). In addition to expert clinical evaluation, autistic traits were quantified by administration of the Autism Quotient

- Yale

Autism Spectrum Disorders (ASD) Participants with ASD were required to meet cutoffs for ASD on the ADOS and (for those who received it) the ADI-R, administered by a research-reliable clinician. They were further required to have a DSM-IV-TR diagnosis of ASD as confirmed by one of three expert clinicians at the Yale Child Study Center, each with 5 or more years of experience in diagnosing ASD, and a combined 45 years of diagnostic experience.

# Information of the subjects

In addition to the information provided in Table 1, we provide the age and sex information of the subjects from the three ASD subtypes. The average age, standard deviations, and the numbers of males and females of each site and each subtype are provided in Table S1.

# Performance under Controlling for Eye Status and Site Difference

To test the performance of tensor decomposition and the results of t-test while controlling for eye status and site difference, we excluded data with closed eyes. In addition, site effects on dynamic functional connectivities, amplitude of low-frequency fluctuation (ALFF), fractional ALFF (fALFF), and gray matter volume (GMV) were further removed using the ComBat harmonization method (Johnson et al., 2007; Fortin et al., 2017, 2018) in Python, which was demonstrated to perform multi-site effect correction for neuroimaging. Information of the new testing dataset is presented in Table S2. Compared with Table S1, it can be found that 24 subjects’ data were excluded and the whole dataset from SBL was removed, making 145 autism, 45 Asperger’s and only 20 PDD-NOS. Based on the new dataset, we performed brain pattern extraction and statistical comparison as described in the Method section. The brain patterns extracted from the new dataset are shown in Figure S1. Because of the reduction in data, the ability of the tensor decomposition method to find brain patterns is affected. The optimal pattern numbers for each subtype decreased, with two patterns for autism, three patterns for Asperger’s and three patterns for PDD-NOS. Compared with the patterns shown in Figure 2, the existing patterns in Figure S1 show a high degree of similarity to the original patterns with the same dominant sub-networks. In addition, new Pattern 2 of autism, new Pattern 3 of Asperger’s, and new Pattern 3 of PDD-NOS are still patterns correlated with the VN, which is consistent with the results in the manuscript. With the exclusion of eye-closed data, this result further confirmed the robustness and effectiveness of the tensor decomposition method. In summary, the tensor decomposition-based brain pattern extraction adopted in this study is a relatively stable pattern extraction method. With an increase in data size, brain pattens and subnetworks will be more precise.

The results of t-test for ALFF, fALFF and GMV are shown in Figures S2, S3, and S4, respectively. The comparison of GMV still showed limited differences among the three subtypes. However, the results of ALFF and fALFF showed differences compared to the comparison based on the original dataset. Comparing Figure S2 with Figure 3, and Figure S3 with Figure 4, it can be seen that the comparisons between autism and Asperger’s remain a high degree of similarity in the two datasets (before and after eye-status control), while the comparisons involving PDD-NOS (autism vs. PDD-NOS and PDD-NOS vs. Asperger’s) showed significant changes. Considering the condition of the brain pattern and comparisons between autism and Asperger’s, we believe that the changes in the comparisons involving PDD-NOS come from the great reduction of PDD-NOS data, where about 29% of the subjects’ data are lost. In other words, the comparison result between Autism and Asperger’s is preserved, even after data reduction. This result illustrates that in the comparison of the three ASD subtypes, the lack of data has a more serious impact on the statistical results than the site difference, especially for subtypes with a small data size.

# Reference

Fortin, J.-P., Cullen, N., Sheline, Y. I., Taylor, W. D., Aselcioglu, I., Cook, P. A., et al. (2018). Harmonization of cortical thickness measurements across scanners and sites. *NeuroImage* 167, 104–120. doi: 10.1016/j.neuroimage.2017.11.024

Fortin, J.-P., Parker, D., Tunç, B., Watanabe, T., Elliott, M. A., Ruparel, K., et al. (2017). Harmonization of multi-site diffusion tensor imaging data. *NeuroImage* 161, 149–170. doi: 10.1016/j.neuroimage.2017.08.047

Johnson, W. E., Li, C., and Rabinovic, A. (2007). Adjusting batch effects in microarray expression data using empirical Bayes methods. *Biostatistics* 8, 118–127. doi: 10.1093/biostatistics/kxj037


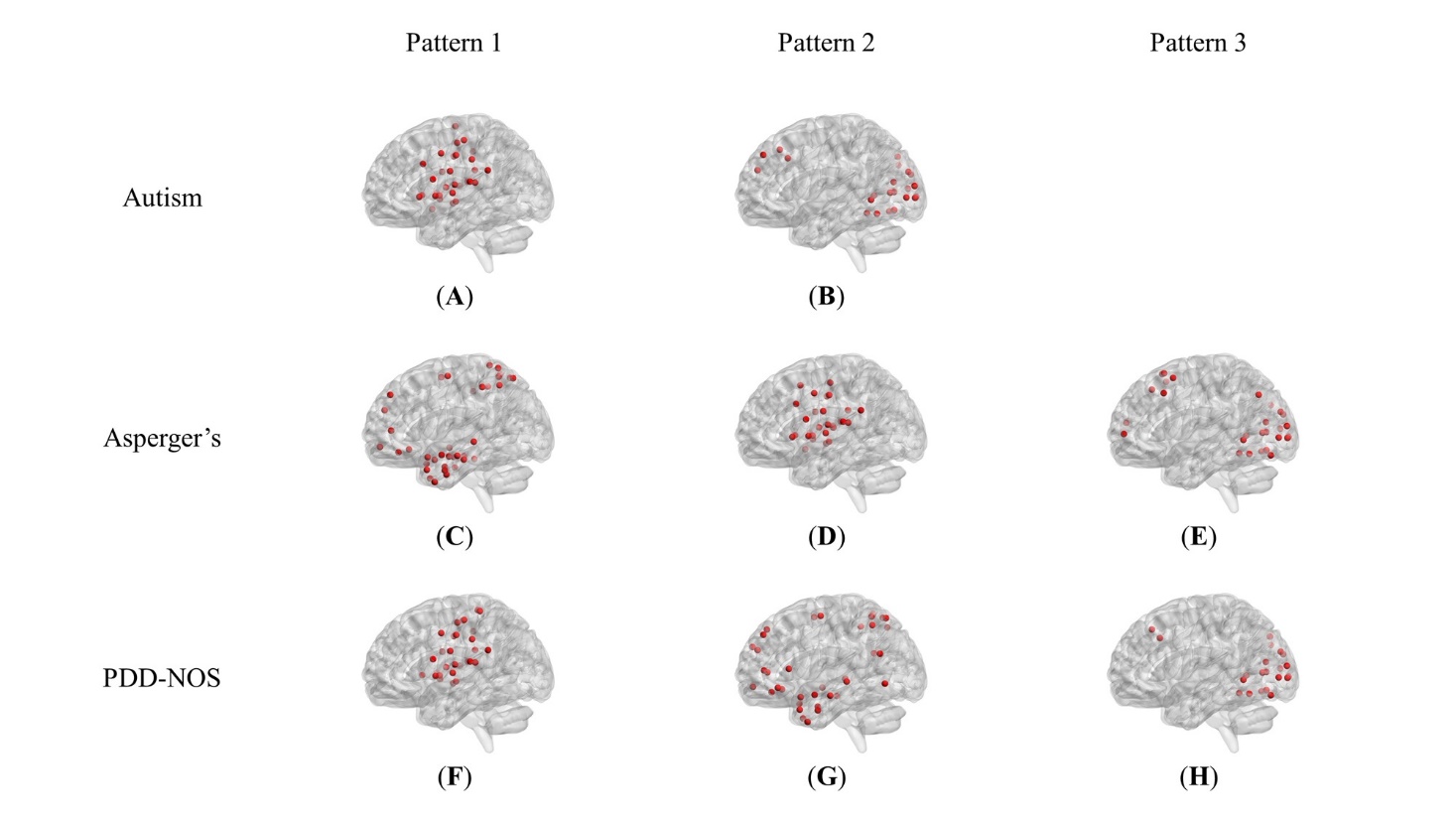


Figure S1. Brain patterns of the ASD subtypes based on the new dataset: (**A**) Pattern 1 of autism (dominated by AN and CON). (**B**) Pattern 2 of autism (dominated by VN). (**C**) Pattern 1 of Asperger’s (dominated by DMN and DAN). (**D**) Pattern 2 of Asperger’s (dominated by AN and CON). (**E**) Pattern 3 of Asperger’s (dominated by VN). (**F**) Pattern 1 of PDD-NOS (dominated by AN, SMH and CON). (**G**) Pattern 2 of PDD-NOS (dominated by DMN and DAN). (**H**) Pattern 3 of PDD-NOS (dominated by VN). AN: auditory network; CON: cingulo-opercular network; SCN: subcortical network; SMM: sensory/somatomotor mouth; SMH: sensory/somatomotor hand; SAN: salience network; FPN: fronto-parietal network; DMN: default mode network; DAN: dorsal attention network; VN: visual network; VAN: ventral attention network; CPN: cingulo-parietal network.


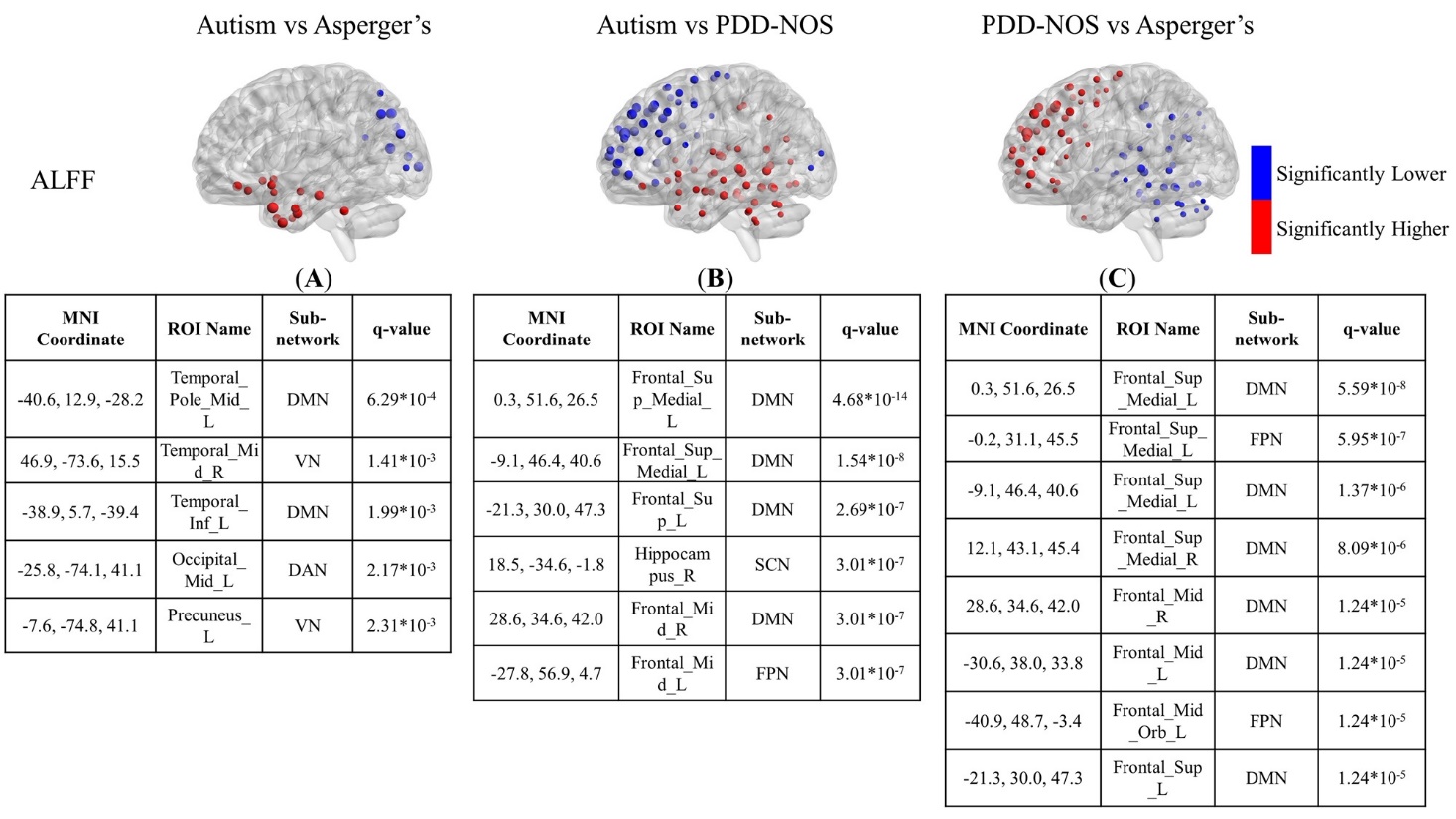


Figure S2. The results of the inter-comparison for ALFF based on the new dataset: (A) is the result of the comparison between autism and Asperger’s; (B) is the result of the comparison between autism and PDD-NOS; and (C) is the result of the comparison between PDD-NOS and Asperger’s. The tables under each subgraph show the ROIs with the top five of the smallest q-values (if there are insufficient ROIs, then all surviving ROIs are shown). It should be noted that the q-values in this study are p-values obtained after FDR correction. The sizes of the ROIs in the figure are related to their q-values. A larger ROI node indicates a smaller q-value, indicating greater significance in the t-test. AN: auditory network; CON: cingulo-opercular network; SCN: subcortical network; SMM: sensory/somatomotor mouth; SMH: sensory/somatomotor hand; SAN: salience network; FPN: fronto-parietal network; DMN: default mode network; DAN: dorsal attention network; VN: visual network; VAN: ventral attention network; CPN: cingulo-parietal network.


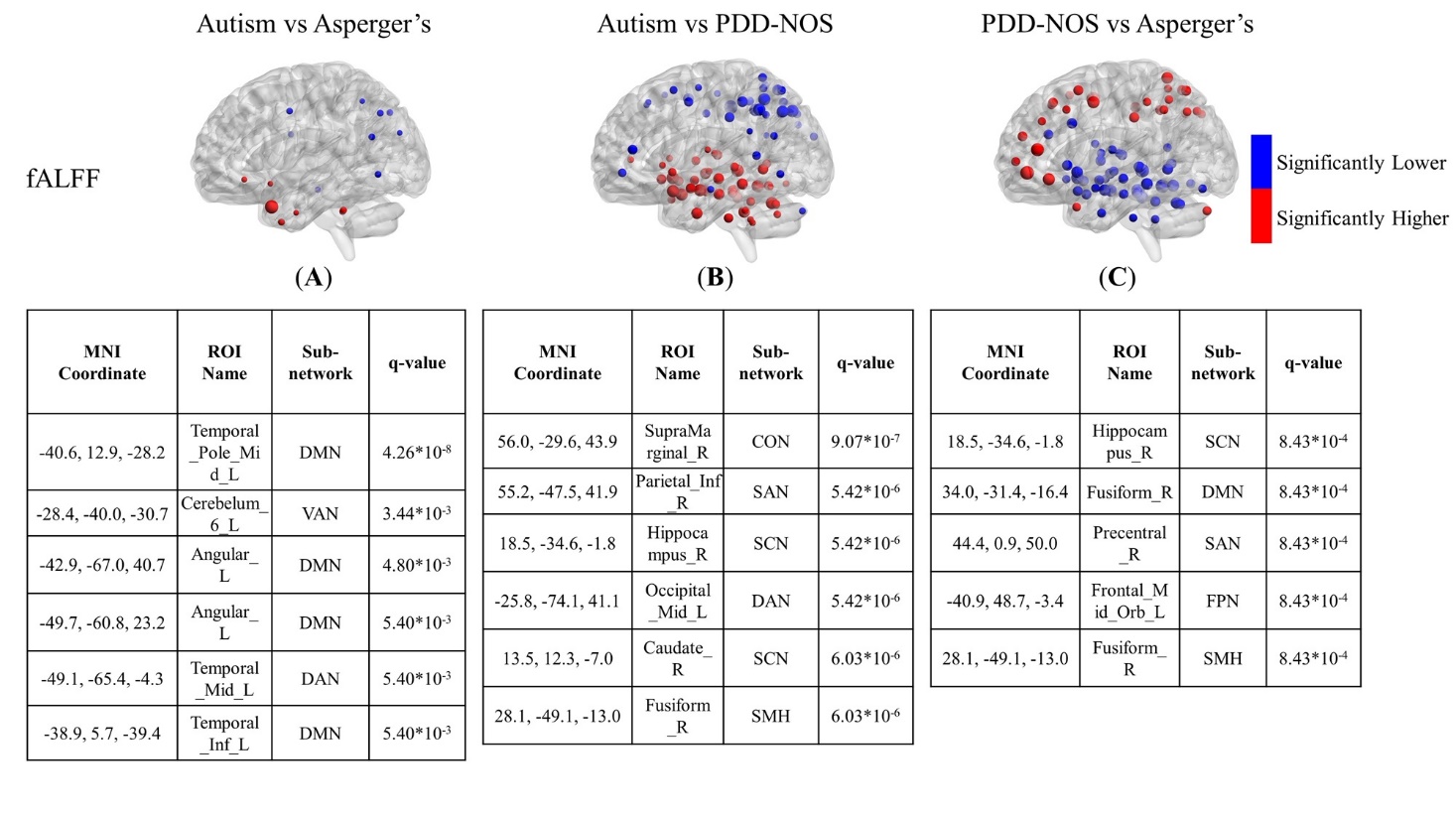


Figure S3. The results of the inter-comparison for fALFF: (A) is the result of the comparison between autism and Asperger’s; (B) is the result of the comparison between autism and PDD-NOS; and (C) is the result of the comparison between PDD-NOS and Asperger’s. The tables under each subgraph show the ROIs with the top five smallest q-values. AN: auditory network; CON: cingulo-opercular network; SCN: subcortical network; SMM: sensory/somatomotor mouth; SMH: sensory/somatomotor hand; SAN: salience network; FPN: fronto-parietal network; DMN: default mode network; DAN: dorsal attention network; VN: visual network; VAN: ventral attention network; CPN: cingulo-parietal network.


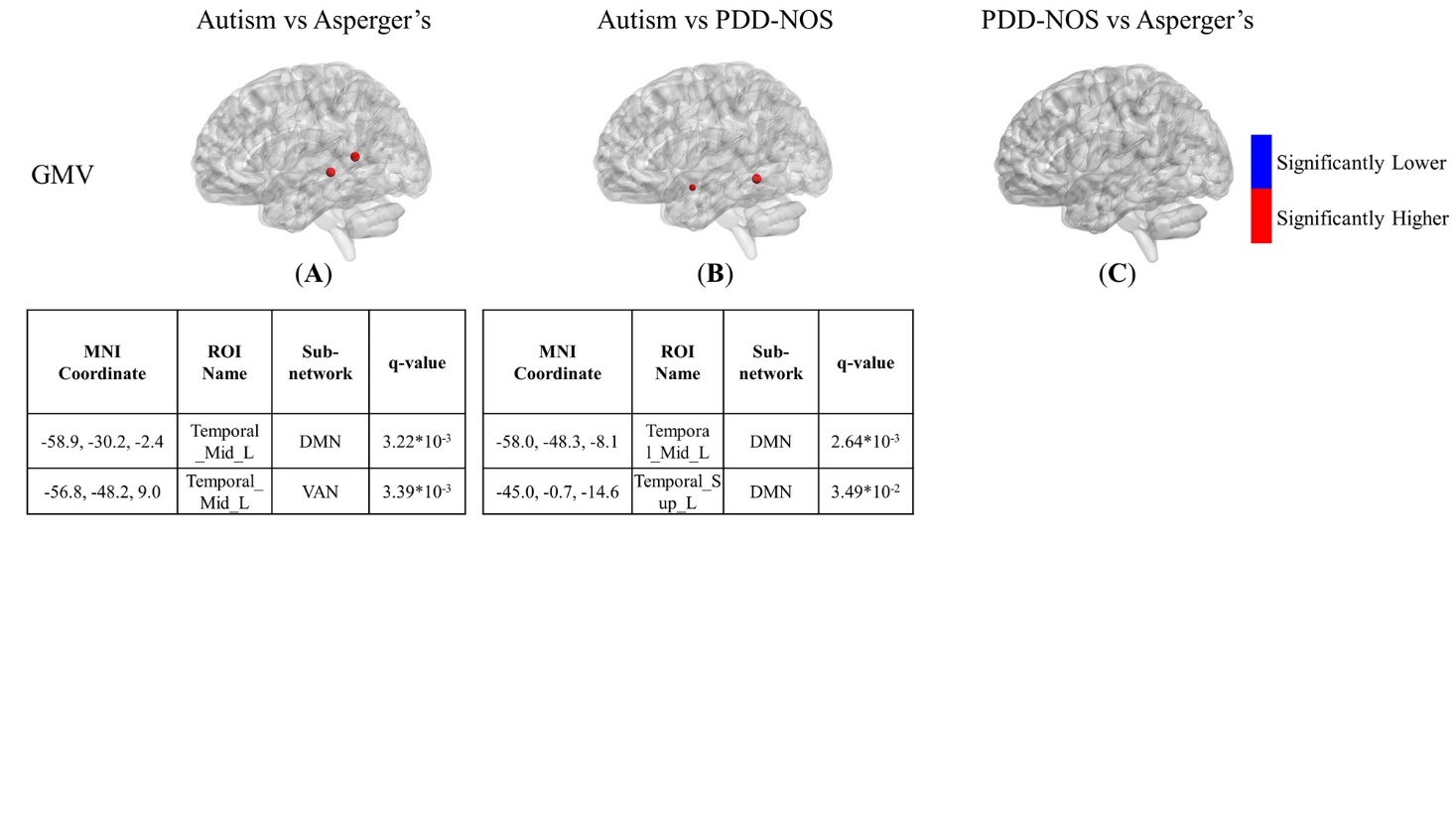


Figure S4. The results of the inter-comparison for GMV: (A) is the result of the comparison between autism and Asperger’s; (B) is the result of the comparison between autism and PDD-NOS; and (C) is the result of the comparison between PDD-NOS and Asperger’s. The tables under each subgraph show the ROIs with the top five smallest q-values. AN: auditory network; CON: cingulo-opercular network; SCN: subcortical network; SMM: sensory/somatomotor mouth; SMH: sensory/somatomotor hand; SAN: salience network; FPN: fronto-parietal network; DMN: default mode network; DAN: dorsal attention network; VN: visual network; VAN: ventral attention network; CPN: cingulo-parietal network.

Table S1. Age and sex information of subjects from the three ASD subtypes.

|  | SITE_ID | AGE_AT_SCAN | Sex (Male/Female) |
| --- | --- | --- | --- |
| Autism | MAX_MUN | 11±0 | 2/0 |
|  | NYU | 13.94±6.32 | 46/6 |
|  | SBL | 27±nan | 1/0 |
|  | SDSU | 13.19±0.87 | 2/1 |
|  | UM_1 | 12.51±2.42 | 31/4 |
|  | UM_2 | 14.92±1.4 | 9/1 |
|  | USM | 22.96±7.43 | 44/0 |
|  | YALE | 15.25±2.16 | 4/1 |
| Autism_Total | - | 16.36±7.1 | 139/13 |
| Asperger’s | MAX_MUN | 18.5±13.9 | 8/0 |
|  | NYU | 18.29±8.66 | 14/4 |
|  | SBL | 32.25±2.22 | 4/0 |
|  | SDSU | 15.26±1.93 | 7/0 |
|  | UM_1 | 12.77±1.9 | 5/1 |
|  | UM_2 | 14.73±2.39 | 3/0 |
|  | YALE | 12.32±3.24 | 6/2 |
| Asperger’s_Total | - | 17.27±8.75 | 47/7 |
| PDD-NOS | NYU | 10.27±2.92 | 5/0 |
|  | SBL | 39.83±12.98 | 6/0 |
|  | SDSU | 15.83±0.33 | 2/0 |
|  | UM_1 | 9.7±nan | 0/1 |
|  | USM | 18.41±nan | 1/0 |
|  | YALE | 11.63±2.77 | 9/4 |
| PDD-NOS_Total | - | 17.9±13.24 | 23/5 |

Table S2. Age and sex information of subjects from the three ASD subtypes (after eye-status control).

|  | SITE_ID | AGE_AT_SCAN | Sex (Male/Female) |
| --- | --- | --- | --- |
| Autism | MAX_MUN | 11.00±0.00 | 2/0 |
|  | NYU | 14.46±6.48 | 41/5 |
|  | SDSU | 13.19±0.87 | 2/1 |
|  | UM_1 | 12.51±2.42 | 31/4 |
|  | UM_2 | 14.92±1.40 | 9/1 |
|  | USM | 22.96±7.43 | 44/0 |
|  | YALE | 15.25±2.16 | 4/1 |
| Autism_Total | - | 16.55±7.08 | 133/12 |
| Asperger’s | MAX_MUN | 10.00±2.00 | 5/0 |
|  | NYU | 16.59±7.14 | 14/2 |
|  | SDSU | 15.26±1.93 | 7/0 |
|  | UM_1 | 12.77±1.90 | 5/1 |
|  | UM_2 | 14.73±2.39 | 3/0 |
|  | YALE | 12.32±3.24 | 6/2 |
| Asperger’s_Total | - | 14.26±5.08 | 40/5 |
| PDD-NOS | NYU | 9.65±0.36 | 3/0 |
|  | SDSU | 15.83±0.33 | 2/0 |
|  | UM_1 | 9.7±nan | 0/1 |
|  | USM | 18.41±nan | 1/0 |
|  | YALE | 11.63±2.77 | 9/4 |
| PDD-NOS_Total | - | 11.99±3.14 | 15/5 |

Table S3. Sub-networks involved in brain patterns. AN: auditory network; CON: cingulo-opercular network; SCN: subcortical network; SMM: sensory/somatomotor mouth; SMH: sensory/somatomotor hand; SAN: salience network; FPN: fronto-parietal network; DMN: default mode network; DAN: dorsal attention network; VN: visual network; VAN: ventral attention network; CPN: cingulo-parietal network.

| ASD Subtype | Brain Pattern | Sub-network (number of ROIs) |
| --- | --- | --- |
| Autism | 1 | **AN(9)**, **CON(8)**, SCN(3), SMM(3), SMH(3), SAN(2) |
|  | 2 | **VN(14)**, DMN(2), FPN(2), VAN(2) |
| Asperger’s | 1 | **DMN(16)**, DAN(6), SMH(3), SCN(3) |
|  | 2 | **AN(8), CON(8)**, SCN(4), SMM(3), SAN(2) |
|  | 3 | **VN(14)**, FPN(4), DMN(3), VAN(3) |
| PDD-NOS | 1 | **AN(9), CON(6), SMH(5)**, SCN(3), SMM(3) |
|  | 2 | **DMN(21), DAN(6)**, VAN(3), FPN(2) |
|  | 3 | **VN(15)**, FPN(2), VAN(2) |
